# Simulation study reveals factors that affect the predominance of SARS-CoV-2 novel variant

**DOI:** 10.1186/s12985-021-01726-6

**Published:** 2021-12-20

**Authors:** Yuki Furuse

**Affiliations:** 1 Nagasaki University Graduate School of Biomedical Sciences, 1-12-4 Sakamoto, Nagasaki, 852-8523 Japan; 2 Medical Education Development Center, Nagasaki University Hospital, 1-7-1 Sakamoto, Nagasaki, 852-8501 Japan; 3 Institute for Frontier Life and Medical Sciences, Kyoto University, 53 Shogoin Kawaharacho, Sakyo-ku, Kyoto, 606-8507 Japan; 4 Hakubi Center for Advanced Research, Kyoto University, Yoshida Honmachi, Sakyo-ku, Kyoto, 606-8501 Japan

**Keywords:** SARS-CoV-2, COVID-19, Epidemiology, Variant, Transmission

## Abstract

The novel variants of the SARS-CoV-2 are a great global concern for the ongoing COVID-19 pandemic. However, how the novel variants predominate and replace existing strains remains elusive. In this study, I simulated the infection spread to investigate what kinds of viral, immunological, and epidemiological factors affect the predominance of SARS-CoV-2 novel variants. The results showed that the increase of the transmissibility of the novel variant substantially enhanced the predominance probability. In addition, the increasing trend of the infection spread, the large case number of the epidemic, and the ability of immune escape of the novel variant increased the predominance probability. A small number of cases and a decreasing trend of an entire epidemic, including not only the novel variant but also earlier strains, are especially important to reduce the chance of the predominance of the novel variant and delay the process. Good control of the COVID-19 epidemic could make the disease burden small and sequester the spread of the SARS-CoV-2 novel variants.

## Introduction

The novel variants of the severe acute respiratory syndrome coronavirus 2 (SARS-CoV-2) keep emerging and hence are a great global concern for the ongoing pandemic of coronavirus disease 2019 (COVID-19). A variant with D614G mutation in the spike protein encoded in the S gene emerged, and its descendants became the major circulating strains in early 2020 possibly due to its high infectivity [1, 2]. The novel variants named Alpha, Beta, and Gamma with characteristic mutations, such as N501Y in the S gene, have emerged and spread worldwide [3]. These novel variants are considered to have high transmissibility, high pathogenicity, and/or the ability to escape from the immunity generated by prior infection with earlier strains or vaccination [4–9].

The emergence and spread of those variants have caused the surge of COVID-19 cases from the end of 2020 to the beginning of 2021 in the United Kingdom, South Africa, and Brazil [5, 10, 11]. The introduction and predominance of those novel variants with the rise of the number of COVID-19 cases were also observed in other parts of the world [12–14]. Many more variants of concern and variants of interest continue to emerge, including the Delta variant, which has even higher transmissibility and has caused a significant impact around the world in 2021 [15]. However, factors that affect the process of the predominance of the novel variants remain elusive.

Here, I performed a simulation study to investigate the causative relationship between viral, immunological, and epidemiological conditions and the process of the predominance of the SARS-CoV-2 novel variants.

## Methods

### Simulation of infection spread

The infection spread was simulated by a stochastic time-discrete compartment model with *S* (susceptible), *I* (infectious), and *R* (removed) compartments in a population of *N* = 1,000,000. On day *t*, the new infections are generated by each infected person on day *t* − 1 according to the reproduction number (*REP*) and the proportion of susceptible people in the population, assuming a negative binomial distribution as follows:

$${I}_{t}={I}_{t-1} +\frac{{ S}_{t-1}}{N} \times \sum_{i=1}^{{I}_{t-1}}rnbinom\left(mean=\frac{REP}{int},\, dispersion=k\right) - \frac{{I}_{t-1}}{int}$$

where *int* indicates the mean serial interval of infection set to 5 days [16] and *k* represents the dispersion parameter of a negative binomial distribution. Heterogeneity in the transmission is determined by *k*; the parameter was set to 0.3 in the simulation according to previous reports on COVID-19 [17, 18].

*I* people move to *R* according to a probability of 1/*int* each day. *R* people are protected from an infection with the already infected strain (either an existing strain or a novel variant). They are also protected from an infection with the other strain on the basis of the degree of cross-immunity described later in the “Methods” section.

Not an infectious period but a serial interval was used for the reproduction of secondary cases. The mean interval between transmission generations (i.e., serial interval) should reproduce the process of infection spread well. Inclusion of the probability density of secondary transmission during an infectious period was not regarded as needed in the model to investigate the predominance process of a novel variant.

Two viral strains were included in the simulation; one is an earlier existing strain, and the other is an emerging novel variant. Five parameters for the precondition in the simulation were investigated. (1) Coefficient to increase the transmissibility of the novel variant (1.0/1.5/2.0). For example, the SARS-CoV-2 Alpha variant was reported to have ~ 1.3–1.5 times higher transmissibility than the original strain. The Delta variant was regarded to have ~ 2.0 times higher transmissibility than the original strain and ~ 1.5 times than the Alpha variant [5, 15]. (2) Epidemic trend defined by reproduction number of the existing strain (0.67/1.0/1.5). For example in Japan, the effective reproduction number of COVID-19 ranges between 0.5 (under a state of emergency) and 2 (when public health interventions were relaxed) [19]. (3) The total number of people infected with the existing strain and the novel variant on day 0 (100/500/2500). 4) Cross-immunity between the existing strain and the novel variant (0.25/0.5/0.7). “0.25” means that only 25% of people infected with the existing strain can develop an immunity to protect them from infection with the novel variant (i.e., the novel variant can escape an immunity made by the earlier strain.) “0.75” means that 75% of people infected with the existing strain become resistant for both the existing strain and the novel variant (i.e., good cross-immunity between the two strains). (5) Prevalence of immuned people in the population on day 0 (0%/40%/80%). Most preexisting immunity was assumed to be generated by vaccination for the existing strain. Cross-protection for the novel variant by vaccination was determined by the cross-immunity parameter aforementioned. The combination of these parameters yielded 243 patterns of preconditions for the simulation.

On day 0, 5% of the infected people were assumed to be infected with the novel variant. Stochastic simulations were run for 200 days by 100 times for each precondition. The predominance of the novel variant was determined when the proportion of people infected with the novel variant surpassed 80% of all infected people. And, the frequency and days for the predominance in the simulations were analyzed for each precondition.

### Data availability

A computer script for the simulations is available at GitHub (https://github.com/yukifuruse1217/variant_simulation).

## Results

I investigated the probability of the SARS-CoV-2 novel variant predominance in the stochastic simulation model exploring conditions that contribute to the process of the predominance. Examined factors included the increase of transmissibility of the novel variant, the trend of the epidemic, the epidemic size including both the existing strain and the novel variant, the cross-immunity between the existing strain and the novel variant, and the prevalence of already immuned people in the population. It should be noted that the present study does not consider a particular variant; rather, I investigated the infection spread dynamics of SARS-CoV-2 novel variants at a general level.

**Fig. 1:**
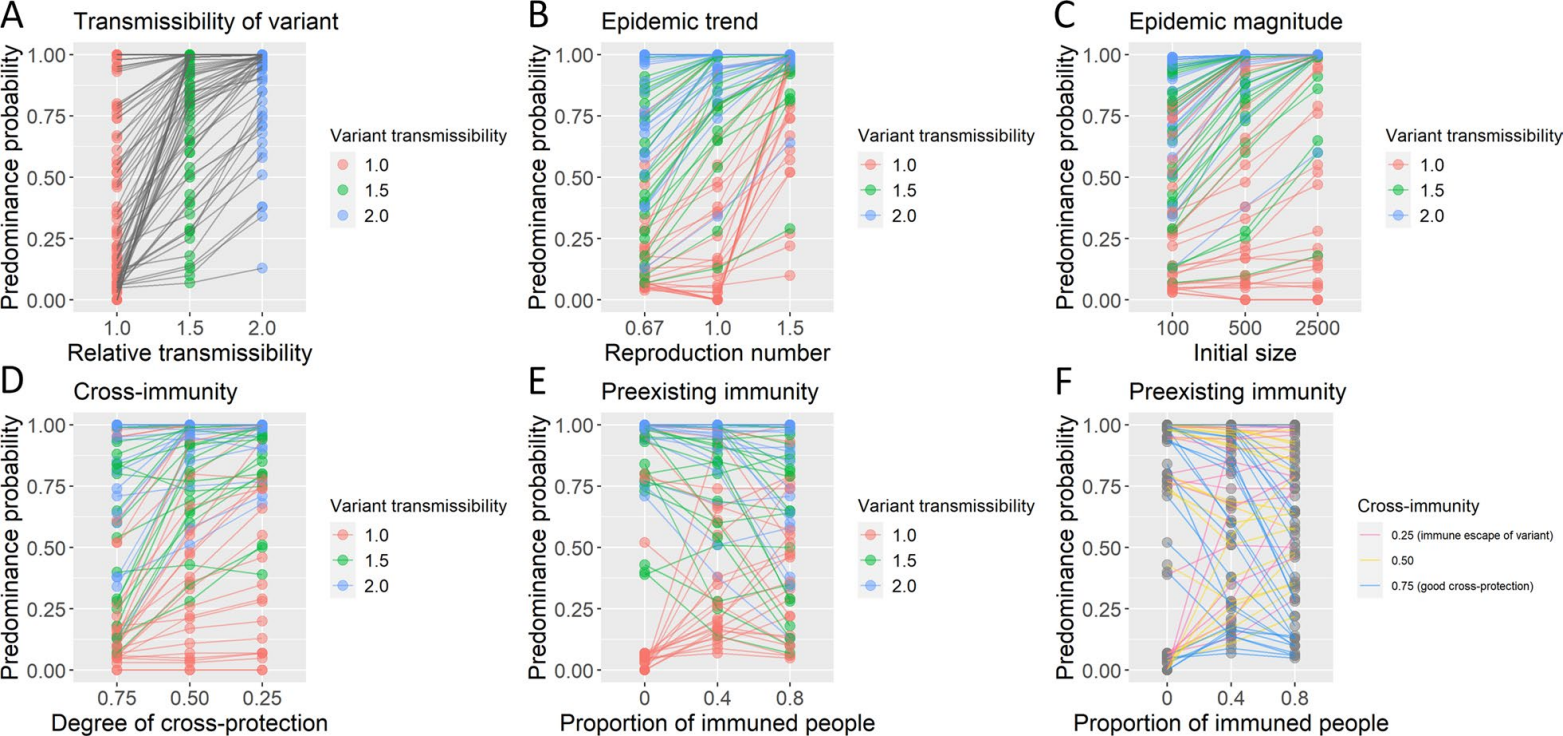
Probability of the predominance of SARS-CoV-2 novel variant by preconditions. The frequencies of predominance in 100 stochastic simulations were plotted for each precondition. The corresponding preconditions with the same parameters, except the variable indicated in the X-axis, were connected with a line. Lines were colored by transmissibility of the novel variant except for panel **F**, in which lines were colored by a parameter of cross-immunity

As expected, the increase of the transmissibility of the novel variant substantially enhanced the predominance probability (Fig. 1A). In addition, the increasing trend of the infection spread of the entire epidemic (i.e., large reproduction number) (Fig. 1B), the large case number of the epidemic (Fig. 1C), and the novel variant’s ability to escape from immunity made by the earlier strain (Fig. 1D) increased the predominance probability.

The proportion of already immuned people showed complicated effects on the predominance probability (Fig. 1E). The high proportion of the preexisting immunity for the earlier strain increased the predominance probability when the novel variant had an immune escape ability (pink lines in Fig. 1F). When the preexisting immunity could prevent the infection with not only the earlier strain but also the novel variant (i.e., good cross-immunity), a higher preexisting immunity then decreased the predominance probability (blue lines in Fig. 1F). There are also Λ-shape patterns in some cases (Fig. 1E, F), suggesting that ~ 40% vaccination coverage could have a higher predominance probability of the novel variant than a very low or very high coverage scenario.

**Fig. 2:**
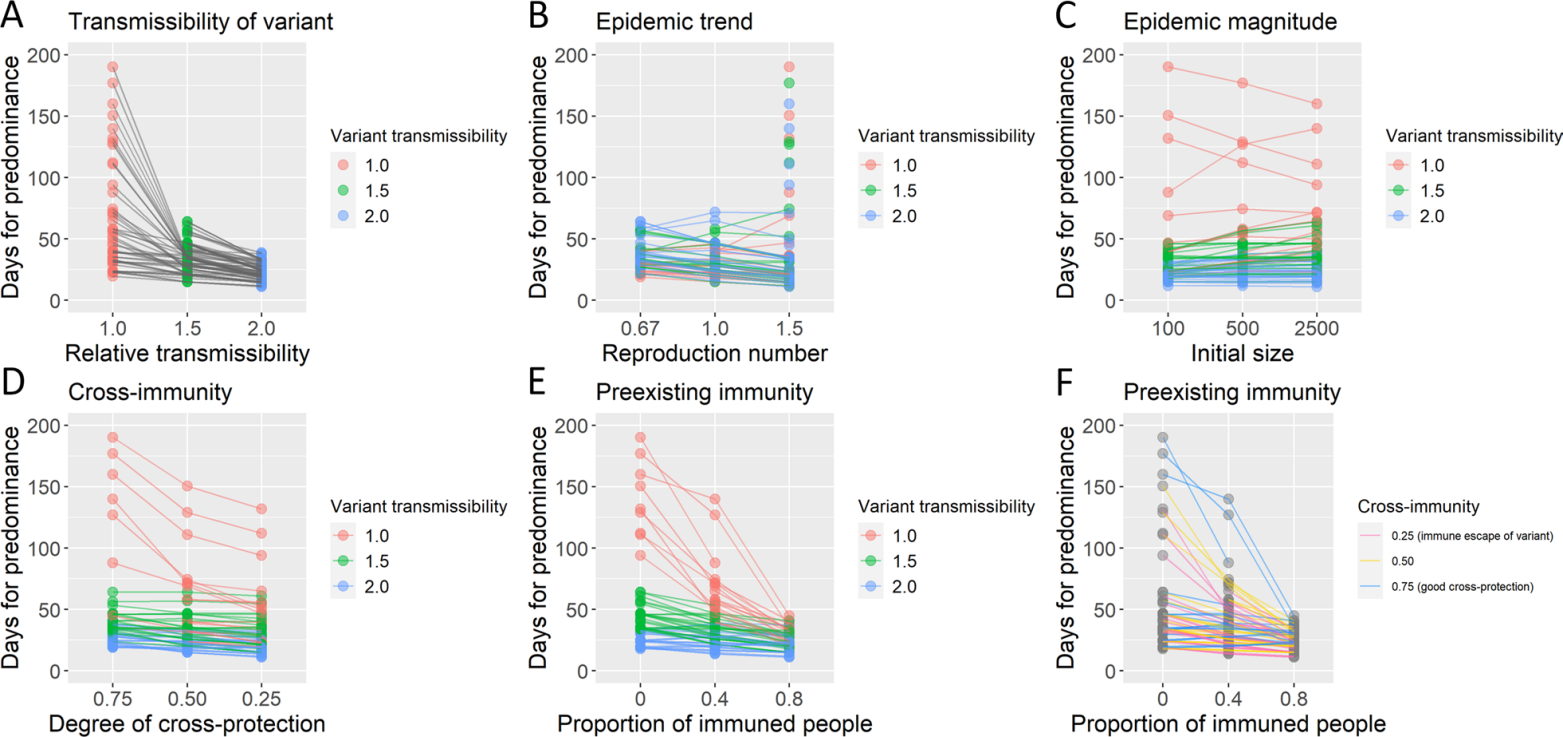
Days for the predominance of SARS-CoV-2 novel variant by preconditions. The median days for the novel variant to predominate in 100 stochastic simulations were plotted for each precondition. The data of simulations with preconditions in which the predominance frequency was less than 20% were excluded from the figure. The corresponding preconditions with the same parameters, except the variable indicated in the X-axis, were connected with a line. Lines were colored by transmissibility of the novel variant except for panel **F**, in which lines were colored by a parameter of cross-immunity

The simulations also found that those factors affect the duration for the novel variant to achieve predominance (Fig. 2). Especially, the increase of the transmissibility of the novel variant (Fig. 2A), the weak cross-immunity between the earlier strain and the novel variant (Fig. 2D), and the high proportion of already immuned people (Fig. 2E, F) shortened the predominance process.

Moreover, it is interesting that the novel variant could predominate even when the variant did not have increased transmissibility (i.e., variant transmissibility = 1.0, red dots in Fig. 1). In contrast, the novel variant could fail to predominate even with two-fold transmissibility (i.e., variant transmissibility = 2.0. blue dots in Fig. 1), especially when the entire epidemic size was small and decreasing. When the number of infected people and reproduction number were small (i.e., initial size = 100 and reproduction number = 0.67. blue dots in Fig. 1B, C), the predominance probability of the novel variant with two-fold transmissibility substantially dropped for some occasions.

## Discussion

Overall, good control of the ongoing COVID-19 epidemic, represented by a small number of cases and its decreasing trend of the entire epidemic including existing and novel strains, can reduce the predominance probability of the SARS-CoV-2 novel variants. Even a variant with no increase in transmissibility can predominate when the epidemic is growing and the variant is able to escape from immunity generated by earlier strains (Fig. 1).

The intervention on the infection spread by public health measures, such as wearing a face mask, physical distancing, rapid case detection, contact tracing, and isolation, is important for not only making the disease burden small but also sequestering the emergence and predominance of the novel variants. Even if it seems impossible to completely stop the spread of the novel variants, we could delay the predominance process. Although the present theoretical study showed the causal relationship between those factors and the predominance of SARS-CoV-2 novel variants, their detailed mechanisms should be further studied in the future.

It is worrisome that the present study found that “halfway” vaccination coverage may increase the predominance probability of novel variants. A previous report also suggested that “intermediate” immune pressure could maximize the possibility of viral adaptation [20]. Therefore, good control of the epidemic will become more crucial to prevent the spread of future variants of SARS-CoV-2 as vaccination is being rolled out.

## Data Availability

A computer script for the simulations is available at GitHub (https://github.com/yukifuruse1217/variant_simulation).

## Abbreviations

COVID-19: Coronavirus disease 2019
SARS-CoV-2: Severe acute respiratory syndrome coronavirus 2
